# Quasi-Entropies and Non-Markovianity

**DOI:** 10.3390/e21101020

**Published:** 2019-10-21

**Authors:** Fabio Benatti, Luigi Brancati

**Affiliations:** 1 Department of Physics, University of Trieste, Strada Costiera 11, I-34151 Trieste, Italy; luigi.brancati93@gmail.com; 2 Istituto Nazionale di Fisica Nucleare (INFN), I-34151 Trieste, Italy

**Keywords:** non-markovian quantum dynamics, dynamical monotones, quasi-entropies

## Abstract

We address an informational puzzle that appears with a non-Markovian open qubit dynamics: namely the fact that, while, according to the existing witnesses of information flows, a single qubit affected by that dissipative dynamics does not show information returning to it from its environment, instead two qubits do show such information when evolving independently under the same dynamics. We solve the puzzle by adding the so-called quasi-entropies to the possible witnesses of information flows.

## 1. Introduction

The theory of non-Markovian quantum dynamics [1,2,3,4] of open quantum systems in interaction with an environment has exposed behaviours that peculiarly differ from those typical of memoryless quantum dynamical semigroups. One possible approach towards non Markovianity is based on the presence of a so-called back-flow of information from environment to system [5,6], while in the standard semigroup setting, information about an open quantum system gets lost to the environment in the course of time.

Within this scheme, there appears an informational puzzle: a recent result [7] provides a non-Markovian qubit dynamics $\Lambda_{t}$ that does not show information flowing from the environment to the system, while the tensor product dynamics $\Lambda_{t}⊗\Lambda_{t}$ does. In other words, in some cases it may happen that while the irreversible time-evolution $\Lambda_{t}$ of one qubit in a given environment is such that information can only flow from the qubit to the environment, by placing two non-interacting qubits in the same environment which affects them independently and in the same way, one instead finds that, under the dynamics $\Lambda_{t}⊗\Lambda_{t}$, information may flow back from the environment to the compound two-qubit open quantum system.

Obviously in order to talk of back-flow of information one needs witnesses to expose it: these witnesses have recently been generically characterised as Physicality Quantifiers [8], namely as functionals $I_{S}^{\left(d\right)}$ on statistical ensembles $E_{S}^{\left(d\right)}$ consisting of *d* quantum states that monotonically decrease under the action of completely positive maps. Within this framework, Markovianity of a time-evolution is identified with the monotonic decrease in time of all possible Physicality Quantifiers $I_{S}^{\left(d\right)}$.

Most Physicality Quantifiers $I_{S}^{\left(d\right)}$ used so far, as trace-distances or generalised trace-distances [5,6], depend on statistical ensembles $E_{S}^{\left(2\right)}$ consisting of two density matrices: all of them are unable to see any back-flow of information under the dynamical maps $\Lambda_{t}$ mentioned above, while they do witness it in the case of $\Lambda_{t}⊗\Lambda_{t}$. On the other hand, Physicality Quantifiers relative to three or more density matrices are scarcely available; therefore, instead of looking for suitable higher degree monotone functionals on $E_{S}^{\left(d\right)}$, with d≥3, we propose to enlarge the class of functionals on $E_{S}^{\left(2\right)}$ by adding to them the so-called quasi-entropies [9,10,11,12]. These quantities, besides generalising to the quantum setting various classical informational tools, may be used in quantum error corrections [12]. Instead, in the following we suggest a dynamical use of them, by showing that quasi-entropies can witness a back-flow of information under $\Lambda_{t}$. The key point will be the lack of Schwartz-Positivity of the maps $\Lambda_{t,s}$ that intertwine the dynamics $\Lambda_{s}$ up to time s≥0, with the dynamics $\Lambda_{t}$ up to time t≥s: $\Lambda_{t}=\Lambda_{t,s}\circ \Lambda_{s}$. In this way, the informational puzzle disappears; namely, it is not true that there is back-flow of information under $\Lambda_{t}⊗\Lambda_{t}$ but not under $\Lambda_{t}$; rather, what occurs is that the back-flow of information due to $\Lambda_{t}$ needs more powerful witnesses to be revealed than those offered so far in the literature.

The material below is organised as follows: in Section 2 we resume the basics of open quantum dynamics and of non-Markovianity by means of a few illustrative models; in Section 3 we introduce the notion of Physicality Quantifiers, present and discuss in detail the informational puzzle relative to the back-flow of information, while in Section 4 we solve the puzzle by means of the quasi-entropies that we then propose to add to the class of Physicality Quantifiers.

## 2. Open Quantum Dynamics

Open quantum systems *S* interacts weakly with their environment *E*, typically a large quantum system with many degrees of freedom in equilibrium with respect to a given Hamiltonian $H_{E}$. The open system *S* is instead a, usually finite, *n*-level system subjected to a Hamiltonian $H_{S}$, while *S* and *E* interact via a Hamiltonian $\lambda H_{SE}$, λ being a small, dimensionless coupling constant making the interaction a perturbation of the free Hamiltonian $H_{S}+H_{E}$.

In the following, we shall mainly focus upon the time-evolution in the Schrödinger picture where the dynamics of the open system *S* is given by linear maps on the state space S(S), that is on the convex set of density matrices, that are n×n positive matrices of trace one, whose eigenvalues are interpreted as probabilities and fix the system *S* statistical properties.

The standard approach [1,2,3,13] to the description of the dynamics of *S* in the presence of the environment *E* starts from a factorised initial state of the systems S+E, namely $\rho_{SE}=\rho ⊗\rho_{E}$, where ρ is any possible system *S* density matrix and $\rho_{E}$ is an environment equilibrium reference state such that $[\rho_{E},H_{E}]=0$. The compound initial state $\rho ⊗\rho_{E}$ evolves in time into $\rho_{SE}\left(t\right)$ according to the Liouville-von Neumann equation

(1) $$\partial_{t}\rho_{SE}\left(t\right)=-i\left[H_{\lambda }\;,\;\rho_{SE}\left(t\right)\right]\;,\;H_{\lambda }=H_{S}+H_{E}+\lambda \;H_{SE}\;,\;\hbar =1\;.$$

The state of *S* at time *t* is obtained by partial tracing over the environment degrees of freedom,

(2) $${\mathrm{Tr}}_{E}\left(\rho_{SE}\right)=\rho ⟼\rho_{t}=\mathrm{Tr}\left(\rho_{SE}\left(t\right)\right)=:\Lambda_{t}\left[\rho \right]\;,$$

yielding a linear dynamical map $\Lambda_{t}$ formally generated by an integro-differential master equation [1,2,3,13]

(3) $$\partial_{t}\rho_{t}=\int_{0}^{t}d\tau \;K\left(t-\tau \right)\left[\rho_{\tau }\right]=\int_{0}^{t}d\tau \;K\left(\tau \right)\left[\rho_{t-\tau }\right]\;,$$

where the kernel K(τ) acts linearly on the state space S(S) and depends on the full Hamiltonian dynamics $e^{-itH_{\lambda }}$ integrated over the past of *S*.

Not much can in general be said about the linear map $\Lambda_{t}$ solution to (3), except that it must be Completely Positive [1], namely that, besides preserving the positivity of any initial state ρ of *S*, when extended to the dynamics $\Lambda_{t}⊗\mathrm{id}$ of the system S+S, that is to the system *S* coupled to an identical inert ancilla *S*, it also preserves the positivity of any density matrix in $M_{n}\left(C\right)⊗M_{n}\left(C\right)$. In the expression above, “id” denotes the identity operation on the algebra $M_{n}\left(C\right)$ of n×n complex matrices associated with the Hilbert space $C^{n}$ of the system *S*.

Dynamical maps $\Lambda_{t}$ must be positivity preserving, in short Positive, as they must turn density matrices into density matrices leaving intact the positivity of their spectrum which must, at each positive time t≥0, amount to a probability distribution. Complete Positivity instead refers to the fact that the extended dynamics $\Lambda_{t}⊗\mathrm{id}$ must preserve the positivity of all states of the compound systems S+S. The reason why Positivity of the map is not enough is that, without Complete Positivity, there might be entangled states of S+S that develop negative eigenvalues under the action of $\Lambda_{t}⊗\mathrm{id}$ [14]. The fact that the solutions of (3) are Completely Positive stems from the maps $\Lambda_{t}$ being expressible in Kraus-Stinespring form [1],

(4) $$\rho_{t}=\Lambda_{t}\left[\rho \right]=\sum_{\alpha }L_{\alpha }\left(t\right)\;\rho \;L_{\alpha }^{†}\left(t\right)\;,\;\sum_{\alpha }L_{\alpha }^{†}\left(t\right)L_{\alpha }\left(t\right)=I,$$

which identifies Completely Positive maps. In the above expression, the matrices $L_{\alpha }\left(t\right)\in M_{n}\left(C\right)$ depend on the whole compound dynamics of open quantum system *S* and environment *E* between times 0 and *t*, while the last equality guarantees the maps $\Lambda_{t}$ to be trace-preserving: $\mathrm{Tr}\left(\rho_{t}\right)=\mathrm{Tr}\left(\rho \right)$ for all t≥0.

### 2.1. Quantum Dynamical Semigroups

Because of its dependence on the system *S* past evolution, the linear map $\Lambda_{t}$ in (4) cannot in general fulfil the forward-in-time semigroup composition law

(5) $$\Lambda_{t}\circ \Lambda_{s}=\Lambda_{s}\circ \Lambda_{t}=\Lambda_{t+s}\;,\;s,t\geq 0\;.$$

Such a property can be enforced enforce by a so-called Markovian approximation whereby in (3) $\rho_{t-\tau }$ is replaced by $\rho_{t}$ and $\int_{0}^{t}d\tau \;K\left(\tau \right)$ by a time independent linear map $L:\rho \mapsto L\left[\rho \right]:=\int_{0}^{+\infty }d\tau \;K\left(\tau \right)$, so that the family of true, but analytically uncontrollable, Completely Positive maps $\Lambda_{t}$, t≥0, solutions to (3), is substituted by a one-parameter semigroup of linear maps $\Lambda_{t}=e^{tL}$ solutions to time-independent master equation

(6) $$\partial_{t}\rho_{t}=L\left[\rho_{t}\right]\;.$$

Only specific approximation procedures like the weak and strong coupling limits and the low density limit [1], guarantee that the approximating maps $\Lambda_{t}$ are Completely Positive: in general, one easily ends up with a not even Positive dynamics [2,3,14,15]. Instead, under the above mentioned mathematically rigorous approximations, the generator L has the so-called GKSL form in agreement with the renown theorems of Gorini-Kossakowski-Sudarshan [16] and Lindblad [17] about the generators of semigroups of Completely Positive, trace-preserving maps $\Lambda_{t}$.

**Theorem** **1.** *A semigroup $\Lambda_{t}$, t≥0, on the state space S(S) of an open quantum system S consists of Completely Positive maps if and only if the generator L reads*

(7) $$L\left[\rho \right]=-i\left[H\;,\;\rho \right]+\sum_{\alpha ,\beta =1}^{n^{2}-1}C_{\alpha \beta }\;\left(F_{\alpha }\;\rho \;F_{\beta }^{†}-\frac{1}{2}\left\{F_{\beta }^{†}\;F_{\alpha }\;,\;\rho \right\}\right)\;,$$

*where the matrices $F_{\alpha }\in M_{n}\left(C\right)$ are traceless and orthogonal with respect to the Hilbert-Schmidt scalar product $\mathrm{Tr}\left(F_{\alpha }^{†}\;F_{\beta }\right)=\delta_{\alpha \beta }$, so that, together with $F_{n^{2}}:=\frac{I}{\sqrt{n}}$, they constitute an orthonormal basis in $M_{n}\left(C\right)$, while the complex coefficients form a Positive semi-definite $\left(n^{2}-1\right)\times \left(n^{2}-1\right)$ (Kossakowski) matrix $C=[C_{\alpha \beta }]\geq 0$.*

The following example discusses the physical consequences of Complete Positivity.

**Example** **1.** *Consider one qubit (2-level system) undergoing a purely dissipative time-evolution generated by the master equation*

(8) $$\partial_{t}\rho_{t}=L\left[\rho \right]=\frac{\alpha }{2}\;\left(\sigma_{1}\;\rho_{t}\;\sigma_{1}-\rho_{t}\right)+\frac{\alpha }{2}\;\left(\sigma_{2}\;\rho_{t}\;\sigma_{2}-\rho_{t}\right)-\frac{\beta }{2}\;\left(\sigma_{3}\;\rho_{t}\;\sigma_{3}-\rho_{t}\right)\;,$$

*where α≥0, with initial condition $\rho_{0}=\rho$. By means of the Pauli matrices $\sigma_{1,2,3}$ and the 2×2 identity matrix I, in the so-called Block representation, one writes the initial density matrix $\rho \in M_{2}\left(C\right)$ as*

(9) $$\rho =\frac{1}{2}\left(I+r_{1}\sigma_{1}+r_{2}\sigma_{2}+r_{3}\sigma_{3}\right)\;,\;r_{1}^{2}+r_{2}^{2}+r_{3}^{2}\leq 1\;,$$

*where the condition on $\vec{r}=\left(r_{1},r_{2},r_{3}\right)\in R^{3}$ is necessary and sufficient for the positivity of the spectrum of ρ. Since $\sigma_{j}\sigma_{k}=\delta_{jk}\;-\;\left(1-\delta_{jk}\right)\;\sigma_{k}\sigma_{j}$, the Pauli matrices are eigenvectors of the generator:*

(10) $$L\left[I\right]=0\;,\;L\left[\sigma_{1}\right]=-\left(\alpha -\beta \right)\;\sigma_{1}\;,\;L\left[\sigma_{2}\right]=-\left(\alpha -\beta \right)\;\sigma_{2}\;,\;L\left[\sigma_{3}\right]=-2\;\alpha \;\sigma_{3}\;.$$

*Then, the semigroup $\Lambda_{t}=e^{tL}$ solution to *(8)* is such that*

(11) $$\Lambda_{t}\left[I\right]=I\;,\;\Lambda_{t}\left[\sigma_{1,2}\right]=e^{-(\alpha -\beta )t}\;\sigma_{1,2}\;,\;\Lambda_{t}\left[\sigma_{3}\right]=e^{-2\alpha t}\sigma_{3}\;,$$

*whence the solution $\rho_{t}=\Lambda_{t}\left[\rho \right]$ to *(8)* reads*

(12) $$\rho_{t}=\frac{1}{2}\;\left(I+e^{-(\alpha -\beta )t}\left(r_{1}\sigma_{1}+r_{2}\sigma_{2}\right)+e^{-2\alpha t}r_{3}\sigma_{3}\right)\;,$$

*whence positivity of $\rho_{t}$ at all times t≥0 is equivalent to α≥β. Whether the maps $\Lambda_{t}$ are also Completely Positive depends on whether the so-called Choi-matrix*

(13) $$\begin{array}{ccc} M_{t} & := & \Lambda_{t}⊗\mathrm{id}\left[P_{sym}\right]=\frac{1}{4}\left(I⊗I+e^{-(\alpha -\beta )t}\left(\sigma_{1}⊗\sigma_{1}-\sigma_{2}⊗\sigma_{2}\right)+e^{-2\alpha t}\sigma_{3}⊗\sigma_{3}\right) \end{array}$$

(14) $$\begin{array}{ccc} & = & \frac{1}{2}\left(\begin{array}{cccc} 1+e^{-2\alpha t} & 0 & 0 & 2e^{-(\alpha -\beta )t}\\ 0 & 1-e^{-2\alpha t} & 0 & 0\\ 0 & 0 & 1-e^{-2\alpha t} & 0\\ 2e^{-(\alpha -\beta )t} & 0 & 0 & 1+e^{-2\alpha t} \end{array}\right)\;, \end{array}$$

*is also positive [18], where*

(15) $$P_{sym}=\frac{1}{4}\left(I⊗I+\sigma_{1}⊗\sigma_{1}-\sigma_{2}⊗\sigma_{2}+\sigma_{3}⊗\sigma_{3}\right)$$

*is the projection onto the symmetric entangled two-qubit state*

(16) $$|\Psi_{sym}\rangle =\frac{1}{\sqrt{2}}\left(|00\rangle +|11\rangle \right)\;,\;\mathrm{where}\;\sigma_{3}|0\rangle =|0\rangle \;,\;\sigma_{3}|1\rangle =-|1\rangle \;.$$

*Notice that $M_{t}$ describes the time-evolution of the initial entangled two-qubit state $P_{sym}$ under the dynamical maps $\Lambda_{t}⊗\mathrm{id}$. One computes that, at small times t≥0, the determinant of $M_{t}$,*

$$Det\left(M_{t}\right)={\left(1-e^{-2\alpha t}\right)}^{2}\left({\left(1+e^{-2\alpha t}\right)}^{2}-4\;e^{-2(\alpha -\beta )t}\right)≃-32\;\alpha^{2}\;\beta \;t^{3}$$

*and can thus be positive if and only if β≤0. The condition α≥β with β>0 guarantees the positivity of $\rho_{t}$, but it makes a negative eigenvalue appear in the spectrum of the time-evolving two-qubit state state $M_{t}$ which could not then be any longer considered a proper density matrix. The condition β≤0 is indeed the only way to comply with the positivity of the Kossakowski matrix $C=\frac{1}{2}\mathrm{diag}\left(\alpha ,\alpha ,-\beta \right)$ associated to the generator in *(8)* and thus, as demanded by Theorem 1, with Complete Positivity.*

Through the positivity of the Kossakowski matrix $C=[C_{\alpha \beta }]$ (see (7)), Complete Positivity puts constraints on the dynamics of the system described by $\Lambda_{t}$; in particular, in the above example the parameter β must be non-positive, while mere Positivity of $\Lambda_{t}$ only asks β not to be larger than α. Complete Positivity is sometimes rejected as an unphysical mathematical simplification [19] because the constraints it imposes upon the dynamics follow from a hypothetical initial statistical coupling of the open quantum system *S* with an uncontrollable, dynamically inert copy of itself whose only role is to allow for initial entanglement between them. A more physically palatable ground for the necessity of Complete Positivity emerges when one considers the dynamical maps $\Lambda_{t}⊗\Lambda_{t}$ on S+S, namely, when the physical context is one where both system and ancilla are under control and in independent weak interaction with a same environment so that they both evolve according to the same map $\Lambda_{t}$. Then, the following result holds [20].

**Theorem** **2.** *$\Lambda_{t}⊗\Lambda_{t}$ is Positive on S(S+S), if and only if $\Lambda_{t}$ is Completely Positive on S(S).*

The physical implications of the above theorem are the following: if $\Lambda_{t}⊗\Lambda_{t}$ is to describe a physical time-evolution, it must be Positive for all t≥0, otherwise negative probabilities may appear in the course of time when one starts with initial bipartite entangled states of S+S. Then, according to the above theorem, necessarily the single system dynamics $\Lambda_{t}$ must be Completely Positive and not only Positive. Notice that if all initial states of S+S were separable, $\rho_{sep}=\sum_{i,j}\lambda_{ij}\rho_{i}^{S}⊗\rho_{j}^{S}$, with $\lambda_{ij}\geq 0$, $\sum_{ij}\lambda_{ij}=1$, and $\rho_{i,j}^{S}\in S\left(S\right)$, then the Positivity of $\Lambda_{t}$ would suffice for physical consistency; indeed,

(17) $$\rho_{sep}\mapsto \rho_{sep}\left(t\right)=\sum_{i,j}\lambda_{ij}\Lambda_{t}\left[\rho_{i}^{S}\right]⊗\Lambda_{t}\left[\rho_{j}^{S}\right]\geq 0\;.$$

This shows that, far from just being a mathematical nicety, Complete Positivity is the dynamical *alter ego* of quantum entanglement.

### 2.2. Non-Markovian Quantum Dynamics

The semigroup composition law (5) follows from the time independence of the generator L in (6) as results from suitable Markovian approximations operated on the integro-differential equation (3). Let us now proceed without such approximations, taking the equation (3) at its face value. Suppose the dynamics $\Lambda_{t}:\rho \mapsto \rho_{t}=\Lambda_{t}\left[\rho \right]$ generated by (3) to be invertible as a linear map, then the time non-local equation can be recast into the time local form

(18) $$\partial_{t}\rho_{t}=\int_{0}^{t}d\tau \;K\left(\tau \right)\circ \Lambda_{t-\tau }\circ \Lambda_{t}^{-1}\left[\rho_{t}\right]=L_{t}\left[\rho_{t}\right]\;,$$

with a time-dependent generator $L_{t}=\int_{0}^{t}d\tau \;K\left(\tau \right)\circ \Lambda_{t-\tau }\circ \Lambda_{t}^{-1}$. Explicitly time-dependent master equations of the form

(19) $$\partial_{t}\rho_{t}=L_{t}\left[\rho_{t}\right]\;,$$

are at the basis of the theory of non-Markovian open quantum dynamics [5,6,21,22,23,24]. Formal solutions to such equations, with initial condition $\rho_{t_{0}}$ at $t=t_{0}\geq 0$ read

(20) $$\rho_{t}=\Lambda_{t,t_{0}}\left[\rho_{t_{0}}\right]:=Te^{\int_{t_{0}}^{t}d\tau \;L_{\tau }}\left[\rho_{t_{0}}\right]\;,$$

by means of the time-ordered exponentials

(21) $$Te^{\int_{t_{0}}^{t}d\tau \;L_{\tau }}:=\mathrm{id}+\sum_{k=1}^{\infty }\int_{t_{0}}^{t}d\tau_{1}\int_{t_{0}}^{\tau_{1}}d\tau_{2}\cdots \int_{t_{0}}^{\tau_{k}}d\tau_{k}\;L_{\tau_{1}}\circ L_{\tau_{2}}\circ \cdots \circ L_{\tau_{k-1}}\;.$$

The maps $\Lambda_{t,t_{0}}$ form a two parameter semigroup:

(22) $$\Lambda_{t,s}\circ \Lambda_{s,t_{0}}=\Lambda_{t,t_{0}}\;\forall \;t\geq s\geq t_{0}\geq 0\;.$$

Setting $\Lambda_{t}:=\Lambda_{t,0}$, the family of dynamical maps $\Lambda_{t}$, t≥0, is automatically *divisible* in the sense that for all t≥s≥0 there exists an intertwining operator mapping $\Lambda_{s}$ into $\Lambda_{t}$; indeed,

(23) $$\Lambda_{t,s}=\Lambda_{t}\circ \Lambda_{s}^{-1}\;,\;\mathrm{and}\;\Lambda_{t}=\Lambda_{t,s}\circ \Lambda_{s}\;\forall \;t\geq s\geq 0\;.$$

**Remark** **1.** *The inverse $\Lambda^{-1}$ of a Completely Positive map* Λ *is Completely Positive if and only if $\Lambda \left[\rho \right]=U\;\rho \;U^{†}$ with U unitary [3]. In general, $\Lambda_{s}^{-1}$ is not Completely Positive and neither is $\Lambda_{t,s}$. However, if $\Lambda_{t,s}$ is Completely Positive for all t≥s≥0 such are also the dynamical maps $\Lambda_{t}=\Lambda_{t,0}$. Nonetheless, as we shall see, the Complete Positivity of $\Lambda_{t}$ for all t≥0 does not require the intertwining maps $\Lambda_{t,s}$ to be Completely Positive. Furthermore, unlike for one-parameter semigroups with time-independent generator, there have not yet been found necessary conditions on the time-dependent generator $L_{t}$, as those provided by Theorem 1, ensuring the Complete Positivity of the generated dynamical maps $\Lambda_{t}$.*

The following theorem fixes the form of the generators $L_{t}$ when they provide Completely Positive intertwining maps $\Lambda_{t,s}$ for all t≥s≥0 [21].

**Theorem** **3.** *The solutions $\Lambda_{t,t_{0}}$ to the time-dependent master equation *(19)*, for all $t\geq t_{0}\geq 0$, are Completely Positive maps if and only if the generator $L_{t}$ reads*

(24) $$L_{t}\left[\rho \right]=-i\left[H_{t}\;,\;\rho \right]+\sum_{\alpha ,\beta =1}^{n^{2}-1}C_{\alpha \beta }\left(t\right)\;\left(F_{\alpha }\;\rho \;F_{\beta }^{†}-\frac{1}{2}\left\{F_{\beta }^{†}\;F_{\alpha }\;,\;\rho \right\}\right)\;,$$

*where the matrices $F_{\alpha }\in M_{n}\left(C\right)$ are as in Theorem 1, while $H_{t}=H_{t}^{†}$ is a time-dependent Hamiltonian and $C_{t}=\left[C_{\alpha \beta }\left(t\right)\right]$ a time-dependent positive semi-definite Kossakowski matrix.*

The previous result states that a positive semi-definite, time-dependent Kossakowski matrix is equivalent to the Complete Positivity of all intertwining maps $\Lambda_{t,s}$, t≥s≥0, associated with $L_{t}$; however, as commented in Remark 1, the latter property is not necessary for the Complete Positivity of $\Lambda_{t}$, t≥0. Therefore, $C_{t}\geq 0$ for all t≥0 implies, but it is not implied by, $\Lambda_{t}$ being Completely Positive for all t≥0.

The following example provides a simple model of qubit dynamics that allows to discuss some of the most prominent aspects of non-Markovianity; in particular, the fact that another property of semigroups fails general one-parameter families of maps $\Lambda_{t}$, t≥0. Namely, unlike in Theorem 2, there exist families of dynamical maps $\Lambda_{t}$ solutions to (19) such that $\Lambda_{t}⊗\Lambda_{t}$ is Positive without $\Lambda_{t}$ being Completely Positive.

**Example** **2.** *Consider the following 1-qubit master equation $\partial_{t}\rho_{t}=L_{t}\left[\rho \right]$ with time-dependent generator [7]:*

(25) $$L_{t}\left[\rho \right]:=\frac{\alpha }{2}\;\left(\sigma_{1}\;\rho_{t}\;\sigma_{1}-\rho_{t}\right)+\frac{\alpha }{2}\;\left(\sigma_{2}\;\rho_{t}\;\sigma_{2}-\rho_{t}\right)-\frac{\alpha }{2}\;\tanh t\;\left(\sigma_{3}\;\rho_{t}\;\sigma_{3}-\rho_{t}\right)\;,\;\alpha \geq 0\;.$$

*By comparison with *(8)*, the generator $L_{t}$ is characterised by a time-dependent Kossakowski matrix*

$$C_{t}=\frac{\alpha }{2}\left(\begin{array}{ccc} 1 & 0 & 0\\ 0 & 1 & 0\\ 0 & 0 & -\tanh t \end{array}\right)$$

*which is Positive semi-definite only at t=0, while −tanht<0 for t>0. Therefore, according to Theorem 3, the intertwining maps $\Lambda_{t,s}$ can never be Completely Positive; nevertheless, the generated maps $\Lambda_{t}$ are Completely Positive only for α≥1 while they are Positive for α≥0. Indeed, as in Example 1,*

(26) $$\begin{array}{ccc} L_{t}\left[I\right]=0 & \;\;L_{t}\left[\sigma_{1,2}\right]=-\alpha \left(1-\tanh t\right)\;\sigma_{1,2}\; & L_{t}\left[\sigma_{3}\right]=-2\alpha \;\sigma_{3} \end{array}$$

(27) $$\begin{array}{ccc} \Lambda_{t}\left[I\right]=I & \;\;\Lambda_{t}\left[\sigma_{1,2}\right]=e^{-\alpha t}\cosh^{\alpha }t\;\sigma_{1,2}\; & \Lambda_{t}\left[\sigma_{3}\right]=e^{-2\alpha t}\;\sigma_{3}\;. \end{array}$$

*Such an action corresponds to $\Lambda_{t}$ being expressible very similarly to the Kraus-Stinespring decomposition in *(4)*:*

(28) $$\rho_{t}:=\Lambda_{t}\left[\rho \right]=\sum_{a=0}^{3}\lambda_{a}\left(t\right)\;\sigma_{a}\;\rho \;\sigma_{a}\;,$$

*with coefficients*

(29) $$\lambda_{0}\left(t\right)=\frac{e^{-\alpha t}}{2}\left(\cosh\left(\alpha t\right)+\cosh^{\alpha }t\right),\;\lambda_{1,2}\left(t\right):=\frac{1-e^{-2\alpha t}}{2},\;\lambda_{3}\left(t\right)=\frac{e^{-\alpha t}}{2}\left(\cosh\left(\alpha t\right)-\cosh^{\alpha }t\right)\;.$$

*The maps $\Lambda_{t}$ are trace-preserving for all α≥0; indeed, $\sum_{\alpha =0}^{3}\lambda_{\alpha }\left(t\right)=1$. Furthermore, by means of the convexity of the function logcosht for t≥0 one checks that $\lambda_{3}\left(t\right)$ is negative for all 0<α<1 and Positive if α≥1. Thus, the expression *(28)* reduces to *(4)* and $\Lambda_{t}$ is Completely Positive only for α=0 and α≥1.*

*On the other hand, $\Lambda_{t}$ is Positive for all α≥0; indeed, in the Block representation *(9)*,*

(30) $$\rho_{t}=\frac{1}{2}\left(I+{\vec{r}}_{t}\cdot \vec{\sigma }\right)\;,\;{\vec{r}}_{t}=\left(r_{1}\;e^{-\alpha t}\cosh^{\alpha }t,r_{2}\;e^{-\alpha t}\cosh^{\alpha }t,r_{3}\;e^{-2\alpha t}\right)\;,$$

*whence the Block vector ${\vec{r}}_{t}$ belongs to the unit sphere in $R^{3}$ and $\Lambda_{t}$ is Positive for all t≥0 so that $\rho_{t}$ is a legitimate 1-qubit state for all α≥0.*

*Let us now consider the tensor product dynamics $\Lambda_{t}⊗\Lambda_{t}$ which describes two qubits subjected to identical and independent interactions with their environment. By means of *(28)*, its action on two qubit states $\rho_{12}$ can also be cast in a Kraus-Stinespring-like diagonal form*

(31) $$\Lambda_{t}⊗\Lambda_{t}\left[\rho_{12}\right]=\sum_{a,b=0}^{3}\lambda_{a}\left(t\right)\;\lambda_{b}\left(t\right)\;\sigma_{a}⊗\sigma_{b}\;\rho_{12}\;\sigma_{a}⊗\sigma_{b}\;,$$

*However, because of $\lambda_{3}\left(t\right)$, the coefficients $\lambda_{a}\left(t\right)\;\lambda_{3}\left(t\right)$, a=0,1,2,3, are negative for 0<α<1. Thus, $\Lambda_{t}⊗\Lambda_{t}$ is Completely Positive when and only when α=0 or α≥1, namely when and only when $\Lambda_{t}$ is Completely Positive.*

*Further, a result in [25] ensures that, for trace preserving qubit maps $\Lambda_{t}$, the Positivity of the tensor product maps, $\Lambda_{t}⊗\Lambda_{t}$, on two qubits is equivalent to the Complete Positivity of the squares, $\Lambda_{t}^{2}=\Lambda_{t}\circ \Lambda_{t}$, of the 1-qubit maps. Since from *(27)* it follows that $\Lambda_{t}^{2}$ acts as $\Lambda_{t}$ by changing α into 2α, $\Lambda_{t}⊗\Lambda_{t}$ is Positive for α≥1/2. Thus, for α∈[1/2,1), $\Lambda_{t}$ and $\Lambda_{t}⊗\Lambda_{t}$ are both Positive but not Completely Positive.*

*Finally, one easily computes the algebraic inverse of $\Lambda_{t}$ as a linear map on S(S),*

(32) $$\Lambda_{t}^{-1}\left[\sigma_{1,2}\right]=\frac{e^{\alpha t}}{\cosh^{\alpha }\left(t\right)}\;\sigma_{1,2}\;,\;\Lambda_{t}^{-1}\left[\sigma_{3}\right]=e^{2\alpha t}\;\sigma_{3}\;.$$

*The intertwining maps thus read $\Lambda_{t,s}=\Lambda_{t}\circ \Lambda_{s}^{-1}$ and are such that*

(33) $$\Lambda_{t,s}\left[\sigma_{1,2}\right]=\mu_{t,s}\;\sigma_{1,2}\;,\;\Lambda_{t,s}\left[\sigma_{3}\right]=\lambda_{t,s}\;\sigma_{3}\;,$$

*where*

(34) $$\mu_{t,s}:=e^{-\alpha (t-s)}\frac{\cosh^{\alpha }\left(t\right)}{\cosh^{\alpha }\left(s\right)}={\left(\frac{1+e^{-2t}}{1+e^{-2s}}\right)}^{\alpha }\;,\;\lambda_{t,s}=e^{-2\alpha (t-s)}\;.$$

We have thus seen that, unlike for one-parameter semigroups, $\Lambda_{t}⊗\Lambda_{t}$ can be Positive with $\Lambda_{t}$ being not Completely Positive; however, similarly to Theorem 2, the following results holds for the intertwining maps $\Lambda_{t,s}$ [7].

**Theorem** **4.** *Given a one-parameter family of maps $\Lambda_{t}$, t≥0, with intertwining maps $\Lambda_{t,s}$, t≥s≥0, then the tensor product maps $\Lambda_{t,s}⊗\Lambda_{t,s}$ on S(S+S) are Positive if and only if the maps $\Lambda_{t,s}$ are Completely Positive.*

Theorem 4 has an important consequence in relation to Example 2: it excludes that the tensor products $\Lambda_{t,s}⊗\Lambda_{t,s}$ of the maps in (30) could be Positive for all t≥s≥0 when α∈[1/2,1). Indeed, if $\Lambda_{t,s}⊗\Lambda_{t,s}$ were Positive, then the single system maps $\Lambda_{t,s}$ would be Completely Positive and thus such would be also the single system dynamical maps $\Lambda_{t}$, but this is impossible for the α in the interval considered.

The qubit time-evolution in the previous example is also an instance of a dynamics $\Lambda_{t}$ which is fully legitimate, namely Completely Positive, for α>1, but non-Markovian according to the so-called *divisibility criterion* that we are now going to discuss (se also Ref. [3,24,26]).

**Definition** **1.** *A one-parameter family $\Lambda_{t}$ of Completely Positive dynamical maps on S(S) is called CP-divisible, respectively P-divisible, if $\Lambda_{t}=\Lambda_{t,s}\circ \Lambda_{s}$ for all t≥s≥0, with Completely Positive, respectively Positive, intertwining maps $\Lambda_{t,s}$. The one-parameter family $\Lambda_{t}$, t≥0, is called Markovian by divisibility if and only if it is CP-divisible.*

Because the generator $L_{t}$ in Example 2 has a non-positive definite Kossakowski matrix, according to Theorem 3, the dynamical maps $\Lambda_{t}$ studied there cannot be CP-divisible. However, they are always P-divisible. Indeed [3,27], trace-preserving maps $\Lambda :M_{n}\left(C\right)\mapsto M_{n}\left(C\right)$ are Positive if and only if they are contractive on self-adjoint operators with respect to the trace norm

(35) $${∥X∥}_{1}:=\mathrm{Tr}\left(\sqrt{X^{†}\;X}\right)\;.$$

For self-adjoint operators $X=X^{†}$, ${∥X∥}_{1}=\mathrm{Tr}\left(X_{+}\right)+\mathrm{Tr}\left(X_{-}\right)$, where $X_{\pm }$ are the positive orthogonal parts of $X=X_{+}\;-\;X_{-}$ and for contractive Λ it holds that

(36) $${∥\Lambda \left[X\right]∥}_{1}\leq {∥X∥}_{1}\;.$$

Then, P-divisibility of invertible maps $\Lambda_{t}$ becomes equivalent to [28]

(37) $$\frac{d}{dt}∥\Lambda_{t}{\left[X\right]∥}_{1}=\frac{d}{dt}{∥\Lambda_{t,s}\circ \Lambda_{s}\left[X\right]∥}_{1}\leq 0\;\forall \;t\geq 0\;,\;\forall X=X^{†}\in M_{n}\left(C\right)\;.$$

This condition is satisfied by the map $\Lambda_{t}$ in the example: indeed, given $X=X^{†}=x_{0}+\sum_{i=1}^{3}x_{i}\sigma_{i}$, using (27) with real $x_{0}$ and $x_{i}$, i=1,2,3, the eigenvalues of $\Lambda_{t}\left[X\right]$ are

(38) $$x_{\pm }\left(t\right)=x_{0}\pm \Delta_{t}\;,\;\Delta_{t}:=\sqrt{x_{3}^{2}e^{-4\alpha t}+\left(x_{1}^{2}+x_{2}^{2}\right)e^{-2\alpha t}\cosh^{2\alpha }\left(t\right)}\;.$$

Since $\Delta_{t}\leq \Delta_{0}$, $∥\Lambda_{t}{\left[X\right]∥}_{1}$ is either constant, $∥\Lambda_{t}{\left[X\right]∥}_{1}=2|x_{0}{|=∥X∥}_{1}$, or it decreases in time, $∥\Lambda_{t}{\left[X\right]∥}_{1}=\;2\;\Delta_{t}\leq \;2\;\Delta_{s}={∥\Lambda_{s}\left[X\right]∥}_{1}$ for t≥s≥0.

On the other hand, in view of the last comment in Example 2, the double tensor products $\Lambda_{t}⊗\Lambda_{t}$ are no longer P-divisible, whence P-divisibilty as much as Positivity is not a property which is in general stable under tensorisation. This is in contrast to Complete Positivity and CP-divisibility: if $\Lambda_{t}$ is Completely Positive or CP-divisible, that is if $\Lambda_{t}=\Lambda_{t,s}\circ \Lambda_{s}$ with $\Lambda_{t,s}$ Completely Positive for all t≥s≥0, then both $\Lambda_{t}⊗\Lambda_{t}$ and $\Lambda_{t,s}⊗\Lambda_{t,s}$ are also Completely Positive. In fact, the Kraus-Sinespring form (4) which identifies Completely Positive maps is robust against tensor products $\Lambda_{t,s}⊗\Lambda_{t,s}$.

The various properties of the maps and intertwining maps introduced in Example 2 are resumed in the following table (Table 1).

## 3. Physicality Quantifiers

The fact that, unlike CP-divisibility, P-divisibility is not stable under double tensorisation represents a puzzle within another approach to non-Markovianity that identifies it with an information back-flow from the environment *E* into the open quantum system *S* [5,6]. In order to discuss this issue, we shall use a recent approach [8] that has been developed as a unifying framework for all measures, called Physicality Quantifiers, of information flows. Indeed, whether the latter goes into the system *S* from the environment *E* or vice versa from *S* into *E* can be witnessed by suitable bounded functionals from all possible statistical ensembles of states of *S* into the positive reals, that monotonically decrease under the action of Completely Positive maps. A statistical ensemble of states of *S* is any set $E_{S}:={\left\{p_{i},\rho_{i}^{S}\right\}}_{i=1}^{n}$ of density matrices $\rho_{i}\in S\left(S\right)$ and statistical weights $p_{i}\geq 0$, $\sum_{i=1}^{n}p_{i}=1$.

**Definition** **2.** *Let $F_{S}^{\left(n\right)}$ denote the set of all statistical ensembles $E_{S}={\left\{p_{i},\rho_{i}^{S}\right\}}_{i=1}^{n}$ comprising n states and weights and let $F_{S}={⋃}_{n=1}^{\infty }F_{S}^{\left(n\right)}$ correspond to the set of all possible statistical ensembles. A Physicality Quantifier, $I_{S}$, is any Positive, bounded functional on $F_{S}$ decreasing under the action of Completely Positive trace-preserving maps* Λ *on the state space of the system S, in the following sense*

(39) $$I_{S}\left(\left\{p_{i},\;\Lambda \left[\rho_{i}\right]\right\}\right)\;\leq \;I_{S}\left(\left\{p_{i},\;\rho_{i})\right\}\right)\;\forall \;t\geq 0\;.$$

The most used among such Physicality Quantifiers are the trace-distance (see (35))

(40) $$D\left(\rho_{1},\rho_{2}\right)=\frac{1}{2}{∥\rho_{1}-\rho_{2}∥}_{1}\;,$$

and the extended trace distance

(41) $$GTD\left(p_{1},p_{2},\rho_{1},\rho_{2}\right):={∥p_{1}\;\rho_{1}\;-\;p_{2}\;\rho_{2}∥}_{1}\;0\leq p_{1}\leq 1\;,\;p_{2}=1-p_{1}\;.$$

Both these Physicality Quantifiers involve two density matrices and thus act on $F_{S}^{\left(2\right)}$ the first one being characterised by fixed weights $p_{1}=p_{2}=1/2$. Both these quantities decrease under Positive (and thus a fortiori under Completely Positive) maps because, as already observed, Positive trace-preserving maps are contractive with respect to the trace-norm.

**Remark** **2.** *Another quantity with the same monotonic behaviour relative to statistical ensembles consisting of two density matrices $\rho_{1,2}$ is the relative entropy*

(42) $$S\left(\rho_{1},\rho_{2}\right)=\mathrm{Tr}\left(\rho_{1}\left(\log\rho_{1}-\log\rho_{2}\right)\right)\;.$$

*This expression vanishes only when $\rho_{1}=\rho_{2}$ and it has been much used in describing entropy production in quantum thermodynamics [29]. Recently, it has been shown [30] that Positivity of $\Lambda_{t}$ is sufficient to ensure that*

(43) $$S\left(\Lambda_{t}\left[\rho_{1}\right],\Lambda_{t}\left[\rho_{2}\right]\right)\leq S\left(\rho_{1},\rho_{2}\right)\;\forall \;t\geq s\geq 0\;.$$

*Though the relative entropy, being unbounded on $F_{S}^{\left(2\right)}$, is not a Physicality Quantifier as in Definition 2, we will nevertheless consider it as such by virtue of its monotonic behaviour.*

All the three previous functionals on $F_{S}^{\left(2\right)}$ measure the degree of distinguishability of two density matrices and can thus be related to how the information about the system *S* behaves in time: the argument goes as follows. In the case of unitary time-evolutions, both the maps $\rho \mapsto \rho_{t}=U_{t}\rho U_{t}^{†}$ and their inverse maps $\rho \mapsto U_{t}^{†}\rho U_{t}$ are of the Kraus-Stinespring form (4) and thus Completely Positive. Therefore, any Physicality Quantifier dos not change in time under their action:

(44) $$I_{S}\left(\left\{p_{i},\;U_{t}\rho_{i}\;U_{t}^{†}\right\}\right)\leq I_{S}\left(\left\{p_{i},\;\rho_{i}\right\}\right)=I_{S}\left(\left\{p_{i},\;U_{t}^{†}U_{t}\rho_{i}U_{t}^{†}U_{t}\right\}\right)\leq I_{S}\left(\left\{p_{i},\;U_{t}\rho_{i}U_{t}^{†}\right\}\right)\;.$$

Given a monotonically decreasing functional $I_{S}$ on $E_{S}=\left\{p_{i},\rho_{i}\right\}$, one may distinguish between an internal information content relative to the systems *S*, only; namely,

(45) $$I_{\mathrm{int}}\left(t\right):=I_{S}\left(\left\{p_{i},\;\Lambda_{t}\left[\rho_{i}\right]\right\}\right)\;,$$

and a global one concerning the entire closed compound system S+E together with its statistical ensembles of the form $\left\{p_{i},\rho_{i}⊗\rho_{E}\right\}$, the environment state $\rho_{E}$ being the equilibrium state from which, together with $U_{t}$, one derives the reduced dynamics $\Lambda_{t}$ of the open system *S*. By subtracting from the global information content the internal one, one gets a measure of the information external to *S*:

(46) $$I_{\mathrm{ext}}\left(t\right):=I_{S+E}\left(\left\{p_{i},\;U_{t}\rho_{i}⊗\rho_{E}U_{t}^{†}\right\}\right)-I_{S}\left(\left\{p_{i},\;\Lambda_{t}\left[\rho_{i}\right]\right\}\right)\;.$$

Since the dynamics of S+E is unitary, $I_{S+E}\left(\left\{p_{i},\;U_{t}\rho_{i}⊗\rho_{E}U_{t}^{†}\right\}\right)$ is constant in time; then,

(47) $$I_{\mathrm{int}}\left(t\right)+I_{\mathrm{ext}}\left(t\right)=I_{S+E}\left(\left\{p_{i},\;\rho_{i}⊗\rho_{E}\right\}\right)\;.$$

Therefore, if $I_{\mathrm{int}}\left(t\right)$ decreases, the information content of the environment increases; this is an effect that can be interpreted as a flow of information from the system *S* to the environment *E*. Vice versa, if $I_{\mathrm{ext}}\left(t\right)$ decreases, the increase of the internal information is interpreted as an information back-flow from the environment to the system. This argument is at the basis of the definition of non-Markovianity proposed in [5], respectively [6], and based on the trace-distance (40), respectively generalised trace-distance (41). Both these approaches to non-Markovianity can be accommodated within the framework of Physicality Quantifiers in [8] where one can introduce various degrees of Markovianity.

**Definition** **3.** *A physical map $\Lambda_{t}$ is said to be $I_{S}$-Markovian if $I_{S}$ is monotonically decreasing in time for any given statistical ensemble $E_{S}\in F_{S}$,*

(48) $$I_{S}\left(\left\{p_{i},\;\Lambda_{t}\left[\rho_{i}\right]\right\}\right)\;\leq \;I_{S}\left(\left\{p_{i},\;\Lambda_{s}\left[\rho_{i}\right])\right\}\right)\;\forall \;t\geq s\geq 0\;.$$

*$\Lambda_{t}$ is said to be n-Markovian if all Physicality Quantifiers $I_{S}$ on $F_{S}^{\left(n\right)}$ are monotonically decreasing and S-Markovian if it is n-Markovian for all n.*

The idea behind this classification is clear: CP-divisible dynamics cannot break the monotonic decrease of Physicality Quantifiers, by the very definition of these latter quantities. However, lack of Markovianity is not identified with lack of CP-divisibility; indeed, as we have seen, the trace-distances and the relative entropy behave monotonically also under the action of P-divisible, but not necessarily CP-divisible maps. Rather, lack of Markovianity is identified with back-flow of information as witnessed by the lack of monotonicity of some Physicality Quantifier of a certain degree that might be higher than that of the trace-distance or of the generalised trace-distance and might thus require statistical ensembles comprising more than two density matrices.

An important result is the following one which asserts that if the generalised trace-distance monotonically decreases under $\Lambda_{t}$ then so must do all Physicality Quantifiers over statistical ensembles with two density matrices.

**Theorem** **5.** *A qubit dynamics $\Lambda_{t}$ is Markovian with respect to the generalised trace-distance if and only if it is 2-S-Markovian.*

Consider the maps $\Lambda_{t}$ in Example 2, they are P-divisible; therefore, because of their contractive character, both the trace-distance and the generalised trace-distance monotonically decrease under they action and thus they and the relative entropy as well signal no back-flow of information. According to Theorem 5, back-flow of information, if any, cannot be witnessed, at the single qubit level, by Physicality Quantifiers in $F_{S}^{\left(2\right)}$. However, the dynamics $\Lambda_{t}⊗\Lambda_{t}$ of two independent qubits is Positive, but not P-divisible for α∈[1/2,1) and can thus be accompanied by back-flow of information as explicitly shown in the following example. The puzzling fact that the information flow can be inverted by tensorisation will be dealt with in the next section.

**Example** **3.** *Let us consider the intertwining map $\Lambda_{t,s}$ in *(32)*. For sufficiently small 0≤Δt, it is linearly approximated by*

(49) $$\Lambda_{t+\Delta t,t}\left[I\right]=I\;,\;\Lambda_{t+\Delta t,t}\left[\sigma_{1,2}\right]≃\sigma_{1,2}-\;\alpha \;\Delta t\;\left(1-\tanh t\right)\;\sigma_{1,2}\;,\;\Lambda_{t+\Delta t,t}\left[\sigma_{3}\right]≃\sigma_{3}\;-\;2\;\alpha \;\Delta t\;\sigma_{3}\;.$$

*Then, up to first order in Δt, its action on the entangled bipartite state $P_{sym}$ in *(15)* yields*

(50) $$Q\left(t,\Delta t\right):=\Lambda_{t+\Delta ,t}⊗\Lambda_{t+\Delta t,t}\left[P_{sym}\right]≃P_{sym}\;-\;\frac{\alpha }{2}\Delta t\;\left(1-\tanh t\right)\;\left(\sigma_{1}⊗\sigma_{1}-\sigma_{2}⊗\sigma_{2}\right)\;-\;\alpha \;\Delta t\;\sigma_{3}⊗\sigma_{3}\;.$$

*It can be checked that, for sufficiently small 0≤Δt and all t>0, Q(t,Δt) has a negative eigenvalue; indeed,*

(51) $$Q\left(t,\Delta t\right)\;\frac{|00\rangle -|11\rangle }{\sqrt{2}}\;≃\;-\alpha \;\Delta t\;\tanh t\;\frac{|00\rangle -|11\rangle }{\sqrt{2}}\;.$$

*Therefore, since $\mathrm{Tr}\left(Q(t,\Delta t)\right)=\mathrm{Tr}\left(P_{sym}\right)=1$, the sum of the absolute values of the eigenvalues of Q(t,Δt), namely its trace-norm, must exceed 1, whence*

(52) $$\begin{array}{ccc} {∥Q(t,\Delta t)∥}_{1} & = & {∥\Lambda_{t+\Delta t}⊗\Lambda_{t+\Delta t}\left[\Lambda_{t}^{-1}⊗\Lambda_{t}^{-1}\left[P_{sym}\right]\right]∥}_{1}\\ & \geq & 1=∥P_{sym}{∥}_{1}={∥\Lambda_{t}⊗\Lambda_{t}\left[\Lambda_{t}^{-1}⊗\Lambda_{t}^{-1}\left[P_{sym}\right]\right]∥}_{1}\;. \end{array}$$

*Consider the trace-norm $N_{t}\left(s\right)$ of*

(53) $$\begin{array}{ccc} \Lambda_{s}⊗\Lambda_{s}\left[\Lambda_{t}^{-1}⊗\Lambda_{t}^{-1}\left[P_{sym}\right]\right] & = & \frac{1}{4}\left(I⊗I\;+\;\mu_{s,t}^{2}\;\left(\sigma_{1}⊗\sigma_{1}-\sigma_{2}⊗\sigma_{2}\right)\;+\;\lambda_{s,t}^{2}\;\sigma_{3}⊗\sigma_{3}\right)\\ & = & \frac{1}{4}\left(\begin{array}{cccc} 1+\lambda_{s,t}^{2} & 0 & 0 & 2\;\mu_{s,t}^{2}\\ 0 & 1-\lambda_{s,t}^{2} & 0 & 0\\ 0 & 0 & 1-\lambda_{s,t}^{2} & 0\\ 2\;\mu_{s,t}^{2} & 0 & 0 & 1+\lambda_{s,t}^{2} \end{array}\right) \end{array}$$

**Figure 1:**
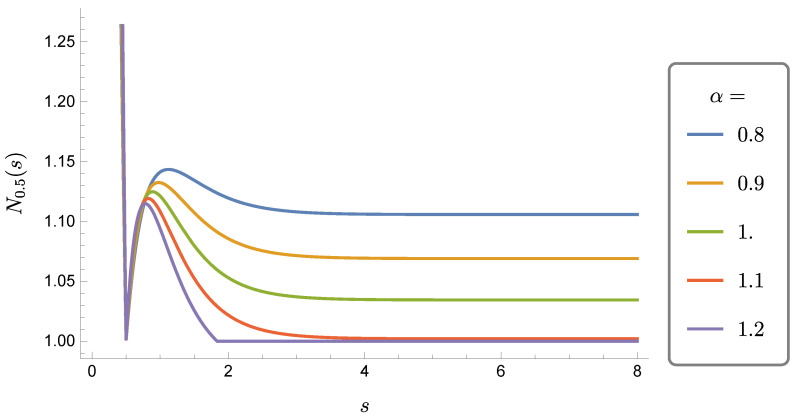
Behaviour of the trace norm of the matrix (53), $N_{t}\left(s\right)$, for t=0.5 and α={0.8,0.9,1.0,1.1,1.2}.

*with $\mu_{s,t}$ and $\lambda_{s,t}$ are as in *(34)* with t and s exchanged. As a function of s≥0 for fixed t, $N_{t}\left(s\right)$ increases for t≤s≤t+Δt, thus revealing the presence of a back-flow of information from the environment to the compound system S+S. The non-monotonically decreasing behaviour of $N_{t}\left(s\right)$ is shown in Figure 1 for various values of α and fixed t=0.5.*

In the following we shall provide a cure to the puzzle presented by the fact that $\Lambda_{t}$ does not show back-flow of information while $\Lambda_{t}⊗\Lambda_{t}$ does. Such a puzzle occurs for 1/2≤α<1, range of values for which $\Lambda_{t}$ is Positive but not Completely Positive: for this range of values, the physical consistency of $\Lambda_{t}$ does not extend then to the maps $\Lambda_{t}⊗\mathrm{id}$ which fail to be Positive. Nonetheless, the maps $\Lambda_{t}⊗\Lambda_{t}$ are Positive and thus represent a physically legitimate two qubit dynamics. Therefore, the informational puzzle cannot be outright discarded as unphysical also in view of the fact that, as already noticed, the available Physicality Quantifiers in $F_{S}^{\left(2\right)}$ monotonically decrease even under maps that are only Positive and not Completely Positive.

## 4. Quasi-Entropies

The hierarchy of Markovianity degrees elaborated in [8] suggests an obvious way out of the informational puzzle presented above; namely, it might occur that the back-flow of information affecting $\Lambda_{t}⊗\Lambda_{t}$ also affects $\Lambda_{t}$, though in a way that cannot be exposed by either the trace-distance, the generalised trace-distance or the relative entropy and that demands Physicality Quantifiers of higher degree than n=2. Indeed, as from Theorem 5, no Physicality Quantifier $I_{S}$ with two density matrices can witness any back flow information affecting a qubit non-Markovian dynamics if this cannot be done by the generalised trace-distance.

Then, one should look for Physicality Quantifiers involving more than two density matrices to see whether monotonic decrease fails at a higher level than on statistical ensembles in $F_{S}^{\left(2\right)}$; unfortunately, very few $I_{S}$ on $F_{S}^{\left(n\right)}$, n≥3, are known in the literature. Instead, we propose to enlarge the class of Physicality Quantifiers by adding to them the so-called quasi-entropies originally introduced in [9,10,11,12] that we will now briefly review.

Given two density matrices $\rho_{1}$ and $\rho_{2}\in M{\left(C\right)}_{n}$, with $\rho_{2}$ invertible, their *relative modular operator*$\Delta (\rho_{1}/\rho_{2})$ is the linear operator acting on the algebra $M_{n}\left(C\right)$ as a linear space and defined by

(54) $$M_{n}\left(C\right)∋a\mapsto \Delta_{\rho_{1}.\rho_{2}}\left[a\right]=\rho_{1}\;a\;\rho_{2}^{-1}\;.$$

By introducing the left and right multiplication operators $L_{b}$ and $R_{b}$, $b\in M_{n}\left(C\right)$ such that

(55) $$M_{n}\left(C\right)∋a\mapsto L_{b}\left[a\right]=b\;a\;,\;R_{b}\left[a\right]=a\;b\;,$$

one can write $\Delta_{\rho_{1},\rho_{2}}=L_{\rho_{1}}\;R_{\rho_{2}^{-1}}$. Then, using the spectral decompositions

(56) $$\rho_{1}=\sum_{i=1}^{n}r_{i}^{\left(1\right)}\;P_{i}^{\left(1\right)}\;,\;\rho_{2}=\sum_{i=1}^{n}r_{i}^{\left(2\right)}\;P_{i}^{\left(2\right)}\;,$$

with orthogonal eigen-projections $P_{i}^{\left(1\right)}$ and $P_{j}^{\left(2\right)}$, one obtains the spectral decomposition

(57) $$\Delta_{\rho_{1},\rho_{2}}=\sum_{i,j=1}^{n}\frac{r_{i}^{\left(1\right)}}{r_{j}^{\left(2\right)}}\;L_{P_{i}^{\left(1\right)}}\;R_{P_{j}^{\left(2\right)}}\;.$$

Indeed, $L_{b}\;R_{b}=R_{b}\;L_{b}$ for all $b\in M_{n}\left(C\right)$ and $L_{b}^{2}=L_{b}$, $R_{b}^{2}=R_{b}$ if $b^{2}=b$: this fact guarantees that $L_{P_{i}^{\left(1\right)}}R_{P_{j}^{\left(2\right)}}$ are idempotent. Furthermore, by considering the matrices in $M_{n}\left(C\right)$ as vectors of the linear space $C^{n}⊗C^{n}$ equipped with the Hilbert-Schmidt product, ${\langle a|b\rangle }_{HS}:=\mathrm{Tr}\left(a^{†}\;b\right)$, the adjoint $L_{b}^{†}$ and $R_{b}^{†}$ of the left and right multiplication operators are given by

(58) $${\langle a|L_{b}\left[c\right]\rangle }_{HS}=\mathrm{Tr}\left(a^{†}\;b\;c\right)=\mathrm{Tr}\left({\left(b^{†}\;a\right)}^{†}\;c\right)={\langle L_{b^{†}}\left[a\right]|c\rangle }_{HS}\;.$$

Thus, $L_{b}^{†}=L_{b^{†}}$ and, similarly $R_{b}^{†}=R_{b^{†}}$, so that $L_{P_{i}^{\left(1\right)}}\;R_{P_{j}^{\left(2\right)}}$ are orthonormal projections onto $M_{n}\left(C\right)$ interpreted as a Hilbert space..

**Definition** **4.** *Given $a\in M_{n}\left(C\right)$ and a real function $f:I\subset R^{+}\to R$ continuous on an interval of the positive half-line, the quasi-entropy relative to a and f of two density matrices $\rho_{1}$ and $\rho_{2}$, with the latter one invertible, is [9,10]*

(59) $$S_{f}^{a}\left(\rho_{1}\;,\;\rho_{2}\right):=\mathrm{Tr}\left(\rho_{2}^{1/2}a^{†}f\left(\Delta_{\rho_{1},\rho_{2}}\right)\left[a\rho_{2}^{1/2}\right]\right)=\langle a\sqrt{\rho_{2}}|\;f\left(\Delta_{\rho_{1},\rho_{2}}\right){|\;a\sqrt{\rho_{2}}\rangle }_{HS}\;,$$

*where in the last equality, the Hilbert-Schmidt scalar product has been used.*

By means of the spectral decomposition (57), one writes

(60) $$f\left(\Delta_{\rho_{1},\rho_{2}}\right)=\sum_{i,j=1}^{n}f\left(\frac{r_{i}^{\left(1\right)}}{r_{j}^{\left(2\right)}}\right)\;L_{P_{i}^{\left(1\right)}}\;R_{P_{j}^{\left(2\right)}}\;,$$

whence the quasi-entropies can be expressed in terms of the eigenvalues $r_{1i}$, $r_{2j}$ and eigenvectors $|r_{1i}\rangle$, $|r_{2j}\rangle$ of $\rho_{1}$, respectively $\rho_{2}$; namely,

(61) $$S_{f}^{a}\left(\rho_{1}\;,\;\rho_{2}\right)=\sum_{i,j=1}^{n}\;r_{j}^{\left(2\right)}\;f\left(\frac{r_{i}^{\left(1\right)}}{r_{j}^{\left(2\right)}}\right)\;{\left|\langle r_{1i}|a|r_{2j}\rangle \right|}^{2}\;.$$

The quasi-entropies generalise many well-known quantum informational tools [9,10,11]; observe that they depend on two density matrices, but also on the operator *a*. In this sense, they also generalise the notion of Physicality Quantifier $I_{S}$ of order 2, namely the family defined on $F_{S}^{\left(2\right)}$.

**Example** **4.** *The following ones are among the most noticeable quasi-entropies.*

- *Choosing $f\left(x\right)=x^{\gamma }$, with γ∈[0,1], from *(61)* one obtains the so-called Lieb functional*
  (62) $$S_{\gamma }^{a}\left(\rho_{1}\;,\;\rho_{2}\right)=\mathrm{Tr}\left(a^{†}\rho_{1}^{\gamma }\;a\;\rho_{2}^{1-\gamma }\right)\;.$$
- *Choosing f(x)=xlog(x) and a=I, one recovers the relative entropy* (42)
  (63) $$S_{f}^{I}\left(\rho_{1}\;,\;\rho_{2}\right)=\mathrm{Tr}\left(\rho_{1}\left(\log\rho_{1}-\log\rho_{2}\right)\right)\;.$$
- *Choosing $f_{\gamma }\left(x\right)=\frac{1-x^{\gamma }}{\gamma (1-\gamma )}$, γ∈(0,1), and a=I yields the so-called relative entropies of degree γ:*
  (64) $$S_{f}^{I}\left(\rho_{1},\rho_{2}\right)=\frac{1}{\gamma (1-\gamma )}\;\mathrm{Tr}\left(\rho_{2}\left(1-\rho_{1}^{\gamma }\;\rho_{2}^{-\gamma }\right)\right):=S_{\gamma }\left(\rho_{2},\rho_{1}\right)\;.$$

In relation to the informational puzzle considered in the previous section, the most important property of quasi-entropies is the following. Consider the class of so-called operator monotonically increasing functions; these are positive functions from the real positive half-line, (0,+∞), into $R^{+}$ such that

(65) $$f\left(x\right)\;\leq \;f\left(y\right)\;\forall \;0\leq x\leq y\in M_{n}\left(C\right)\;,$$

where $f\left(x\right)=\sum_{i=1}^{n}f\left(x_{i}\right)\;P_{i}$ if $x=\sum_{i=1}^{n}x_{i}\;P_{i}$, $x_{i}\geq 0$, is the spectral decomposition of x≥0. Then, the quasi-entropies increase under the action of the so-called Schwartz-Positive unital maps $\Lambda^{T}$, namely linear maps from $M_{n}\left(C\right)$ into $\mapsto M_{n}\left(C\right)$ such that $\Lambda^{T}\left[I\right]=I$, $\Lambda^{T}\left[a^{†}\right]={\left(\Lambda^{T}\left[a\right]\right)}^{†}$ and

(66) $$\Lambda^{T}\left[a^{†}\;a\right]\geq \Lambda^{T}\left[a^{†}\right]\;\Lambda^{T}\left[a\right]\;\forall \;a\in M_{n}\left(C\right)\;,$$

where $\Lambda^{T}$ is such that $\mathrm{Tr}\left(\rho \;\Lambda^{T}\left[a\right]\right)=\mathrm{Tr}\left(\Lambda \left[\rho \right]\;a\right)$ for all $a\in M_{n}\left(C\right)$, with Λ trace-preserving on density matrices. Indeed, one has the following result [11], a sketch of whose proof is provided in Appendix A.

**Theorem** **6.** *Let $f:R^{+}\to R$ be a continuous operator monotonically increasing function with f(0)≥0. Then, the quasi entropy corresponding to f is monotonically increasing with respect to to any Schwartz-Positive* Λ, *in the following sense:*

(67) $$S_{f}^{a}\left(\Lambda \left[\rho_{1}\right],\Lambda \left[\rho_{2}\right]\right)\geq S_{f}^{\Lambda^{T}\left[a\right]}\left(\rho_{1},\rho_{2}\right)\;\forall \;a\in M_{n}\left(C\right)$$

*and for all density matrices $\rho_{1},\rho_{2}$ with $\rho_{2}$ invertible.*

Notice that unital Completely Positive-maps Λ are certainly Schwartz-Positive; indeed, they are in particular 2-Positive, whence $\Lambda ⊗{\mathrm{id}}_{2}$ is a Positive linear map on matrices $a⊗I\in M_{n}\left(C\right)⊗M_{2}\left(C\right)$:

$$0\leq \left(\begin{array}{cc} I & a^{†}\\ a & a^{†}a \end{array}\right)\mapsto \left(\begin{array}{cc} I & \Lambda {\left[a\right]}^{†}\\ \Lambda [a] & \Lambda [a^{†}a] \end{array}\right)\geq 0\;.$$

Then, $\Lambda \left[a^{†}a\right]\geq \Lambda {\left[a\right]}^{†}\Lambda \left[a\right]$ follows from

$$\left(\begin{array}{cc} \langle \phi |\Lambda {\left[a\right]}^{†} & -\langle \phi | \end{array}\right)\left(\begin{array}{cc} I & \Lambda {\left[a\right]}^{†}\\ \Lambda [a] & \Lambda [a^{†}a] \end{array}\right)\left(\begin{array}{c} \Lambda [a]|\phi \rangle \\ -|\phi \rangle \end{array}\right)\geq 0\;\forall |\phi \rangle \in C^{n}\;.$$

**Example** **5.** *Consider the map discussed in Example 1, which is Positive for all α≥0 and α≥β, but Completely Positive only when β=0 and set α=β≠0. Then, choosing $a=\sigma_{-}$ one has $\sigma_{+}\sigma_{-}=\frac{I+\sigma_{3}}{2}$ and from (11):*

$$\Lambda_{t}^{T}\left[\sigma_{\pm }\right]=\sigma_{\pm }\;,\;\Lambda_{t}^{T}\left[\sigma_{+}\sigma_{-}\right]=\frac{I+e^{-2\alpha t}\sigma_{3}}{2}\;,\;\Delta \left(t\right):=\Lambda_{t}^{T}\left[\sigma_{+}\sigma_{-}\right]\;-\;\Lambda_{t}^{T}\left[\sigma_{+}\right]\Lambda_{t}^{T}\left[\sigma_{-}\right]=-\frac{1-e^{-2\alpha t}}{2}\;\sigma_{3}\;.$$

*The matrix Δ(t) has always a negative eigenvalue unless β=0. Therefore, for β≠0, $\Lambda_{t}$ is Positive; however, it fails to be not only Completely Positive but also Schwartz Positive.*

What matters with Physicality Quantifiers is their monotonic decrease under Completely Positive maps as in Equation (48) of Definition 3; therefore, based on the preceding theorem, we here propose to extend the class of Physicality Quantifiers to include also the quasi-entropies defined by matrix monotonically decreasing functions $f:R_{+}\mapsto R$, with f(0)≤0, and such that 0≤x≤y implies f(x)≥f(y). For such functions the result in Theorem 6 reads:

(68) $$S_{f}^{a}\left(\Lambda_{t}\left[\rho_{1}\right],\Lambda_{t}\left[\rho_{2}\right]\right)\leq S_{f}^{\Lambda_{t}^{T}\left[a\right]}\left(\rho_{1},\rho_{2}\right)\;\forall \;a\in M_{n}\left(C\right)$$

for all dynamical maps $\Lambda_{t}$, t≥0, on S(S) with Schwartz-Positive duals $\Lambda_{t}^{T}$ on $M_{n}\left(C\right)$.

Including the quasi-entropies in the class of Physicality Quantifiers will extend the constraints on any purportedly Markovian dynamics $\Lambda_{t}$ that must then also fulfil monotonic decrease:

(69) $$S_{f}^{a}\left(\Lambda_{t}\left[\rho_{1}\right],\Lambda_{t}\left[\rho_{2}\right]\right)\leq S_{f}^{\Lambda_{t,s}^{T}\left[a\right]}\left(\Lambda_{s}\left[\rho_{1}\right],\Lambda_{s}\left[\rho_{2}\right]\right)\;\forall \;t\geq s\geq 0\;,$$

with respect to all quasi-entropies satisfying (68), where $\Lambda_{t,s}^{T}$ is the dual of the intertwining operator such that $\Lambda_{t}=\Lambda_{t,s}\circ \Lambda_{s}$. Notice that, with respect to the monotonic behaviour (48) of the standard Physicality Quantifiers, in the above inequality, beside the density matrices $\rho_{1,2}$ there also appears the operator *a* at the left hand side which at the right hand side has evolved in time to $\Lambda_{t,s}^{T}\left[a\right]$ under the action of the dual intertwiner.

**Example** **6.** *The function $f_{\epsilon }\left(x\right)=-\log\left(\frac{x+\epsilon }{\epsilon }\right)$ is matrix monotonically decreasing and f(0)=0, then choosing a=I, the expression *(61)* yields (notice the exchange, with respect to Definition 4, of the density matrices in the argument)*

$$S_{f_{\epsilon }}^{I}\left(\rho_{2},\rho_{1}\right)=-\sum_{ij}r_{1j}\log\left(r_{2j}+\epsilon \;r_{1i}\right)-\log\epsilon +\sum_{i}r_{1i}\log r_{1i}\;,$$

*where $\rho_{1}$ is assumed to be invertible or the inverse $\rho_{1}^{-1}$ is defined on the range of $\rho_{1}$ [10]. Then, for all unital, Schwartz-Positive maps $\Lambda^{T}:M_{n}\left(C\right)\mapsto M_{n}\left(C\right)$, $\Lambda^{T}\left[I\right]=I$, Theorem 6 yields (with the monotonically increasing function f turned into the monotonically decreasing function −f)*

(70) $$S_{f_{\epsilon }}^{I}\left(\Lambda \left[\rho_{2}\right],\Lambda \left[\rho_{1}\right]\right)\leq S_{f_{\epsilon }}^{I}\left(\rho_{2},\rho_{1}\right)\;.$$

*Then, the limit ϵ→0 recovers the monotonicity of the relative entropy *(42)* under dynamical maps* Λ *that are dual of unital, Schwartz-Positive ones (compare Remark 2), this latter result being now a particular case of the more general monotonicity of the relative entropy under maps that are dual of unital, only Positive ones [30].*

*Instead, consider the maps $\Lambda_{t}$ discussed in Examples 1 and 5 which are not Schwartz-Positive for β≠0 and choose*

$$a=\sigma_{-}=\frac{\sigma_{1}-i\sigma_{2}}{2}\;,\;\rho_{1}=\frac{I+r\sigma_{3}}{2}\;,\;\rho_{2}=\frac{I}{2}\;,$$

*with −1≤r≤1. Then, as $\Lambda_{t}^{T}\left[\sigma_{\pm }\right]=\sigma_{\pm }$, one gets*

$$S_{f_{\epsilon }}^{\sigma_{-}}\left(\Lambda_{t}\left[\rho_{2}\right],\frac{I}{2}\right)=-\frac{1}{2}\log\frac{1+\epsilon -e^{-2\alpha t}\;r}{\epsilon }\;,\;S_{f_{\epsilon }}^{\Lambda_{t}^{T}\left[\sigma_{-}\right]}\left(\rho_{2}\right],\frac{I}{2})=-\frac{1}{2}\log\frac{1+\epsilon \;-\;r}{\epsilon }\;,$$

*whence, for r≥0,*

$$\Delta \left(t\right):=S_{f_{\epsilon }}^{\sigma_{-}}\left(\Lambda_{t}\left[\rho_{2}\right],\frac{I}{2}\right)\;-\;S_{f_{\epsilon }}^{\Lambda_{t}^{T}\left[\sigma_{-}\right]}\left(\rho_{2},\frac{I}{2}\right)=\frac{1}{2}\log\frac{1+\epsilon \;-\;r}{1+\epsilon -e^{-2\alpha t}\;r}\geq 0$$

*and the monotonic decrease of the quasi-entropy for t≥0 is broken.*

### Back-Flow of Information

Having enlarged the class of Physicality Quantifiers by the introduction of the quasi-entropies, there is a wider spectrum of quantities whose monotonic decrease must be guaranteed in order to ensure Markovianity, namely absence of back-flow of information. We shall now show that to the lack of *P*-divisibility of $\Lambda_{t}⊗\Lambda_{t}$, with $\Lambda_{t}$ as in Example 2, and thus to the breaking of Markovianity by back-flow of information due to the tensor product dynamics, there indeed corresponds a back-flow of information also at the level of the single system dynamics $\Lambda_{t}$. Such a back-flow of information is invisible to Physicality Quantifiers as the trace distances and the relative entropy, but exposable by suitable quasi-entropies. In view of Theorem 6, such a witnessing is possible only if the intertwining map $\Lambda_{t,s}$ are not Schwartz-Positive.

As generalised Physicality Quantifier we consider the quasi entropy (62) built upon inverting the sign of the function $f_{\gamma }\left(x\right)=-x^{\gamma }$, 0≤γ≤1, and upon choosing

(71) $$a=\sigma_{+}:=\frac{\sigma_{1}+i\sigma_{2}}{2}\;,\;\rho_{1}=\rho =\frac{I+r\sigma_{3}}{2}\;,\;\rho_{2}=\frac{I}{2}\;.$$

Using the expressions (27) for the action of $\Lambda_{t}$ and (33) for the action of the intertwining map $\Lambda_{t,s}$ one sees that it coincides with its dual and computes

(72) $$\Lambda_{t}\left[\rho_{1}\right]=\rho \left(t\right)=\frac{I+\lambda_{t}\;r\;\sigma_{3}}{2}\;,\;\Lambda_{t}\left[\rho_{2}\right]=\frac{I}{2}\;,\;\Lambda_{t,s}^{T}\left[\sigma_{+}\right]=\mu_{t,s}\;\sigma_{+}\;,$$

where $\mu_{t}:=\mu_{t,0}$ and $\lambda_{t}:=\lambda_{t,0}$ are given in (34). Then, from (62) with $x^{\gamma }$ changed into $-x^{\gamma }$, one computes

(73) $$S_{\gamma }^{\sigma_{+}}\left(\rho \left(t\right),\frac{I}{2}\right)=-\frac{1}{2}{\left(1+\lambda_{t}\;r\right)}^{\gamma }\;,\;S_{\gamma }^{\Lambda^{T}\left[\sigma_{+}\right]}\left(\rho \left(s\right),\frac{I}{2}\right)=-\frac{1}{2}\mu_{t,s}^{2}{\left(1+\lambda_{s}\;r\right)}^{\gamma }\;.$$

In order to see whether the chosen quasi-entropy, as a Physicality Quantifier, can actually witness a back-flow of information due to $\Lambda_{t}$ when $\Lambda_{t}⊗\Lambda_{t}$ exhibits one, namely for α≥1/2, one needs to check the behaviour in time of the sign of the difference

(74) $$\Delta_{\gamma }\left(t,s\right):=S_{\gamma }^{\sigma_{+}}\left(\rho \left(t\right),\frac{I}{2}\right)-S_{\gamma }^{\mu_{t,s}\sigma_{+}}\left(\rho \left(s\right),\frac{I}{2}\right)=\frac{1}{2}\mu_{t,s}^{2}{\left(1+\lambda_{s}\;r\right)}^{\gamma }\;-\;\frac{1}{2}{\left(1+\lambda_{t}\;r\right)}^{\gamma }\;.$$

Whenever $\Delta_{\gamma }\left(t,s\right)$ changes from negative to positive, the chosen quasi-entropy reveals that information starts flowing from the environment into the system.

Due to Theorem 6, the only chance to avoid a monotone behaviour and see a change in sign in between 0≤s≤t is that the intertwining map $\Lambda_{t,s}$ be not Schwarz-Positive on $\sigma_{-}\sigma_{+}$ when α≥1/2: whether and when this occurs can be ascertained by looking at the eigenvalues of the matrix

(75) $$\Lambda_{t,s}\left[\sigma_{-}\sigma_{+}\right]-\Lambda_{t,s}\left[\sigma_{-}\right]\Lambda_{t,s}\left[\sigma_{+}\right]=\frac{1-\lambda_{t,s}\;\sigma_{3}}{2}-\mu_{t,s}^{2}\frac{1-\sigma_{3}}{2}\;,$$

with $\mu_{t,s}$ and $\lambda_{t,s}$ in (34), which are

(76) $$e_{1}\left(t,s\right)=\frac{1-e^{-2\alpha (t-s)}}{2}\geq 0\;,\;e_{2}\left(t,s\right)=\frac{1}{2}\left(1+e^{-2\alpha (t-s)}\left(1-2{\left(\frac{\cosh t}{\cosh s}\right)}^{2\alpha }\right)\right)\;.$$

Lack of Schwarz-Positivity can thus be witnessed by $e_{2}\left(t,s\right)$ becoming negative for some t≥s≥0.

**Figure 2:**
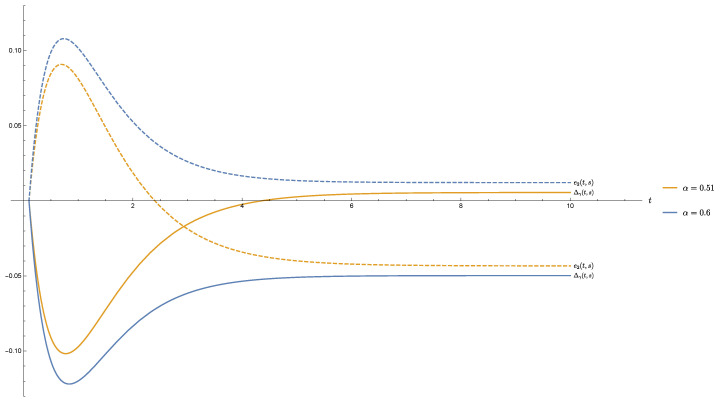
$\Delta_{\gamma }\left(t,s\right)$ (continuous lines) and $e_{2}\left(t,s\right)$ (dashed lines) for r=0.98, γ=0.98, s=0.1 and α={0.51,0.6}.

The following Figure 2 concerns the behaviour of both the quantities $\Delta_{\gamma }\left(t,s\right)$ and $e_{2}\left(t,s\right)$ for the following choices of parameters: r=0.98, γ=0.98 and α=0.51,0.6. It shows the lack of Schwartz-Positivity for α=0.51 which then allows, at a later time, the breaking of monotonic decrease of $\Delta_{\gamma }\left(t,s\right)$. The figure also shows that the monotonic decrease of the chosen quasi-entropy is never broken when Schwartz-Positivity holds, that is for α=0.6.

## 5. Conclusions

We discussed and solved an informational puzzle provided by an example of non-Markovian qubit dynamics $\Lambda_{t}$ which does not show back-flow of information from the environment to the qubit which is instead witnessed when considering the tensor product dynamics $\Lambda_{t}⊗\Lambda_{t}$ of two dynamically non-interacting qubits both embedded within the same environment. The solution to the puzzle consists in enlarging the class of witnesses involving two density matrices by means of the so-called quasi-entropies which are monotonic with respect to Schwartz-Positive maps: a suitable quasi-entropy of Lieb functional type is indeed able to witness the presence of a back-flow of information under $\Lambda_{t}$. The reason is that because the maps $\Lambda_{t}$ are P-divisible maps with intertwining maps $\Lambda_{t,s}$, $\Lambda_{t}=\Lambda_{t,s}\circ \Lambda_{s}$, t≥s≥0, that are Positive but may fail to be Schwartz-Positive.

## Figures and Tables

**Table 1:** Positivity and Complete Positivity of maps, intertwining maps and their tensor products with varying α.

Maps/α	$\Lambda_{t}$	$\Lambda_{t}⊗\Lambda_{t}$	$\Lambda_{t,s}$	$\Lambda_{t,s}⊗\Lambda_{t,s}$
0<α≤1/2	P, not CP	not P	P	not P
1/2≤α≤1	P, not CP	P, not CP	P, not CP	not P
α≥1	CP	CP	P, not CP	not P

## Abbreviations

The following abbreviations are used in this manuscript: CP standing for Completely Positive

MDPI	Multidisciplinary Digital Publishing Institute
DOAJ	Directory of open access journals
TLA	Three letter acronym
LD	linear dichroism

## Appendix A. Quasi-Entropy Monotonicity

We provide here a sketch of the proof, taken from [9,10] that, as in Theorem 6,

$$S_{f}^{a}\left(\Lambda \left[\rho_{1}\right],\Lambda \left[\rho_{2}\right]\right)\geq S_{f}^{\Lambda^{T}\left[a\right]}\left(\rho_{1},\rho_{2}\right)\;\forall \;a\in M_{n}\left(C\right)$$

for all monotonically increasing functions *f*, $a\in M_{n}\left(C\right)$ and for all density matrices $\rho_{1},\rho_{2}$ with $\rho_{2}$ invertible.

It proves convenient to work within the Hilbert-Schmidt framework where matrices $a\in M_{n}\left(C\right)$ become vectors and the relative modular operator a linear operator acting on them. Let us rewrite

(A1) $$\begin{array}{ccc} S_{f}^{\Lambda^{T}\left[a\right]}\left(\rho_{1},\rho_{2}\right) & = & {\langle \;\Lambda^{T}\left[a\right]\sqrt{\rho_{2}}\left|\;f\left(\Delta_{\rho_{1},\rho_{2}}\right)\right|\;\Lambda^{T}\left[a\right]\sqrt{\rho_{2}}\rangle }_{HS} \end{array}$$

(A2) $$\begin{array}{ccc} & = & {\langle \;a\sqrt{\Lambda [\rho_{2}]}\left|\;V^{†}\;f\left(\Delta_{\rho_{1},\rho_{2}}\right)\;V\right|\;a\sqrt{\Lambda [\rho_{2}]}\rangle }_{HS}\;, \end{array}$$

where *V* is a linear operator sending vectors of the form $\left|{a\sqrt{\Lambda [\rho_{2}]}\rangle }_{HS}\right.$, with varying $a\in M_{n}\left(C\right)$, into vectors of the form $\left|\left.\Lambda^{T}\left[a\right]\sqrt{\rho_{2}}\rangle_{HS}\right.\right.$. Due to the assumed Schwartz-Positivity of the map $\Lambda^{T}$ on $M_{n}\left(C\right)$, one gets

$$\begin{array}{cc} & \;{\langle \;a\sqrt{\Lambda [\rho_{2}]}\left|\;V^{†}\;\Delta_{\rho_{1},\rho_{2}}\;V\;\right|\;a\sqrt{\Lambda [\rho_{2}]}\rangle }_{HS}={\langle \;\Lambda^{T}\left[a\right]\sqrt{\rho_{2}}\left|\;\Delta_{\rho_{1},\rho_{2}}\;\right|\;\Lambda^{T}\left[a\right]\sqrt{\rho_{2}}\rangle }_{HS}=\mathrm{Tr}\left(\rho_{1}\Lambda^{T}\left[a^{†}\right]\;\Lambda^{T}\left[a\right]\right)\\ & \;\leq \mathrm{Tr}\left(\rho_{1}\;\Lambda^{T}\left[a^{†}a\right]\right)=\mathrm{Tr}\left(\Lambda \left[\rho_{1}\right]\;a^{†}\;a\right)={\langle \;a\sqrt{\Lambda [\rho_{2}]}\left|\;\Delta_{\Lambda \left[\rho_{1}\right],\Lambda \left[\rho_{2}\right]}\;\right|\;a\sqrt{\Lambda [\rho_{2}]}\rangle }_{HS}\;, \end{array}$$

whence $V^{†}\;\Delta_{\rho_{1},\rho_{2}}\;V\;\leq \;\Delta_{\Lambda \left[\rho_{1}\right],\Lambda \left[\rho_{2}\right]}$. Furthermore, again because of Schwartz-Positivity, the operator *V* is contractive with respect to the Hilbert-Schmidt norm:

(A3) $$\begin{array}{c} {\langle \;a\sqrt{\Lambda [\rho_{2}]}\left|\;V^{†}\;V\right|\;a\sqrt{\Lambda [\rho_{2}]}\rangle }_{HS}=\mathrm{Tr}\left(\rho_{2}\Lambda^{T}\left[a^{†}\right]\Lambda^{T}\left[a\right]\right)\\ \;\leq \mathrm{Tr}\left(\rho_{2}\Lambda^{T}\left[a^{†}\;a\right]\right)=\mathrm{Tr}\left(\Lambda \left[\rho_{2}\right]a^{†}a]\right)={\langle \;a\sqrt{\Lambda [\rho_{2}]}|\;a\sqrt{\Lambda [\rho_{2}]}\rangle }_{HS}\;. \end{array}$$

Then, since f(x) is matrix-monotone, if f(0)=0, then it s also matrix convex, whence

(A4) $$V^{†}\;f\left(\Delta_{\rho_{1},\rho_{2}}\right)\;V\;\leq \;f\left(V^{†}\Delta_{\rho_{1},\rho_{2}}V\right)\leq f\left(\Delta_{\Lambda \left[\rho_{1}\right],\Lambda \left[\rho_{2}\right]}\right)\;.$$

Finally from (A1) and (A2) one deduces

(A5) $$\begin{array}{ccc} S_{f}^{\Lambda^{T}\left[a\right]}\left(\rho_{1},\rho_{2}\right) & = & {\langle \;a\sqrt{\Lambda [\rho_{2}]}\left|\;V^{†}\;f\left(\Delta_{\rho_{1},\rho_{2}}\right)\;V\right|\;a\sqrt{\Lambda [\rho_{2}]}\rangle }_{HS}\\ & \leq & {\langle \;a\sqrt{\Lambda [\rho_{2}]}\left|\;f\left(\Delta_{\Lambda \left[\rho_{1}\right],\Lambda \left[\rho_{2}\right]}\right)\right|\;a\sqrt{\Lambda [\rho_{2}]}\rangle }_{HS}=S_{f}^{a}\left(\Lambda \left[\rho_{1}\right],\Lambda \left[\rho_{2}\right]\right)\;. \end{array}$$
